# Targeting the master regulator mTOR: a new approach to prevent the neurological of consequences of parasitic infections?

**DOI:** 10.1186/s13071-017-2528-3

**Published:** 2017-11-21

**Authors:** Sheila Donnelly, Wilhelmina M. Huston, Michael Johnson, Natalia Tiberti, Bernadette Saunders, Bronwyn O’Brien, Catherine Burke, Maurizio Labbate, Valery Combes

**Affiliations:** School of Life Sciences, Faculty of Science, University of Technology Sydney, Ultimo, NSW 2007 Australia

**Keywords:** mTOR, Rapamycin, Neuropathology, Malaria, Protozoa, Adjunctive therapy, Cerebral Parasitosis

## Abstract

A systematic analysis of 240 causes of death in 2013 revealed that parasitic diseases were responsible for more than one million deaths. The vast majority of these fatalities resulted from protozoan infections presenting with neurological sequelae. In the absence of a vaccine, development of effective therapies is essential to improving global public health. In 2015, an intriguing strategy to prevent cerebral malaria was proposed by Gordon et al. 2015 mBio, 6:e00625. Their study suggested that inhibition of the mammalian target of rapamycin prevented experimental cerebral malaria by blocking the damage to the blood brain barrier and stopping the accumulation of parasitized red blood cells and T cells in the brain. Here, we hypothesize that the same therapeutic strategy could be adopted for other protozoan infections with a brain tropism, to prevent cerebral parasitosis by limiting pathogen replication and preventing immune mediated destruction of brain tissue.

## Background

Protozoan parasites represent a significant threat to human health [1]. Three of the most important diseases caused by protozoan parasites (malaria, trypanosomiasis and toxoplasmosis) are associated with cerebral parasitosis which results in fatalities or leaves survivors with debilitating neurological defects (Table 1) [2]. These diseases contribute to approximately 84 million disability adjusted life years globally, a significant burden which is exacerbated by the lack of licensed vaccines, making safe and effective drugs vital to their prevention and treatment.

**Table 1:** Protozoan parasites with a cerebral tropism

Parasite	Lifestyle	CNS pathological features and severity	Clinical outcome
*Plasmodium falciparum*	Obligate / erythrocytes	Acute cerebral malaria (CM): blood flow obstruction; altered BBB in brain parenchyma [40]	Seizures, retinopathy, elevated cranial pressure leading to brainstem alterations and brain swelling, coma and mortality
*Acanthamoeba*	Free-living	Acute: severe oedema and haemorrhagic necrosis, severe meningeal irritation and encephalitis; amoebic cysts found in brain [23]	Headache, fever, altered state of consciousness, loss of reflex activity, abnormal speech and motor patterns. *Acanthamoeba* granulomatous encephalitis fatal in 90% of cases
*Trypanosoma brucei rhodesiense*	Free-living / blood, lymph, CSF	Acute: severe complications including leptomengitis, early meningo-encepahlitis and encephalitis	Sleep cycle disturbance, headache, tremors, loss of motor control, fatal if left untreated
*Trypanosoma brucei gambiense*	Free-living / blood, lymph, CSF	Chronic: complications that develop more slowly are accompanied by *T. B. gambiense*	Sleep cycle disturbance, headache, tremors, loss of motor control, fatal if left untreated
*Toxoplasma gondii*	Obligate / any nucleated cell	Latent infection characterised by cyst formation in the brain parenchyma [26]. The acute stage is characterised by rapid tachyzoite proliferation in the brain parenchyma. Reactivation of a chronic infection can occur in the immunocompromised [26]	The chronic stage is generally asymptomatic, but the infection can reactivate if the patient becomes immunosuppressed. The acute stage is characterised by headache, epilepsy, hemiparesis, psychosis, cognitive impairment or adynamia

*Abbreviations*: *BBB* Blood brain barrier, *CSF* Cerebrospinal fluid

This review examines the potential use of inhibitors of the mammalian target of rapamycin (mTOR) as adjunctive therapy in the treatment of protozoan cerebral parasitosis and explores the limitations of such approaches by considering the function of mTOR in both the parasite and the host.

## Targeting mTOR: A new strategy to prevent cerebral malaria?

The most severe outcome from infection with the protozoan *Plasmodium falciparum* is human cerebral malaria. This condition is associated with pronounced accumulation of multiple immune cells into the brain [3]. Among these, it is the CD8^+^ T cells that recognise parasite antigens presented, in the context of MHC class1, by parasitised red blood cells (PRBC), and subsequently produce granzyme B and perforin to breakdown tight junctions of the blood brain barrier (BBB) [3]. The increased permeability permits trafficking of inflammatory leukocytes into the brain. It is also these parasite-specific CD8^+^ T cells that modulate the phenotype and function of macrophages, which subsequently secrete damaging pro-inflammatory cytokines.

The murine model of cerebral malaria (experimental cerebral malaria, ECM) has been used for several decades to improve understanding of the disease pathogenesis, and to test new therapies, as it recapitulates most of the features of the paediatric disease, including ataxia, paralysis, coma and death [4, 5]. These common features extend also to post-recovery observations, such as long-term cognitive impairment [6]. However this model has been the subject of many discussions, mainly because pathogenesis of human CM is said to be driven by sequestration of PRBC [7] while ECM is driven by immunological cells such as CD8 T cells and macrophages [8]. It is, however, accepted that PRBC sequestration alone is not sufficient to explain the neuropathology observed during HCM [9]. In fact, recent advances in parasite labelling have demonstrated that PRBC do sequester in microvessels in mice [10] and that PRBC sequestration is indeed a canonical feature of ECM [11, 12]. The recent study by Strangward et al. [12] also showed that ECM recapitulated neuronal damage observed in humans. CD8 T cells are a major mediator of ECM; however, these cells were also identified in post-mortem samples from children who died from CM albeit in small numbers [9]. In a recent review Howland et al. [3] suggested that rather than the presence in mice and absence in human, it was the level of sequestration of immune cells such as CD8 T cells that differed between HCM and ECM with the phenomenon always present and likely relevant. In addition, it is important to note that for understandable reasons, post-mortems studies are only allowing end-point observations, and it is therefore difficult to evaluate the relevance of findings such as the presence of CD8 T cells within the samples. Nonetheless, findings that levels of CXCL-10, a major mediator of CD8 and CD4 T cells migration, allow to differentiate patients with CM from those with severe anaemia and is associated with higher mortality risk, suggests that a non-negligible role can be given to CD8 T cells in the pathogenesis of HCM [13]. Using this model, Gordon et al. [14] identified a potential new adjunctive therapy by targeting the mammalian target of rapamycin (mTOR), a kinase with a central role in maintaining immune homeostasis.

Under steady-state conditions, multiple mechanisms operate in concert to inhibit mTOR expression and/or activity and maintain/restore T cell homeostasis [15]. After the recognition of antigen by naïve T cells, mTOR becomes activated and plays an integral role in the differentiation of CD4^+^ T cells into distinct effector subsets (Th1, Th2, Th17, Tregs and follicular Th cells), and the activation and clonal expansion of CD8^+^ T cells. Thus, mTOR determines T cell fate [15]. In addition, mTOR regulates the function of most other immune cells, including B cells, neutrophils, monocytes/macrophages, dendritic cells, mast cells, and NK cells, making mTOR a central regulator of both innate and adaptive immune responses [16]. Given this breadth of activities, the modulation of mTOR functions was recognised as an attractive therapeutic target, notably in T cell driven immune responses.

Gordon et al. [14] observed an increased survival rate in mice infected with the murine parasite *Plasmodium berghei* ANKA and treated with rapamycin. This was associated with reduced breakdown of the BBB, less haemorrhaging in the brain parenchyma and reduced accumulation of PRBC and leukocytes (notably CD4^+^ and CD8^+^ T cells) within the brain microvasculature. In agreement with these observations, a genome-wide DNA analysis showed that the most affected pathways, both in the brain and the spleen, were associated with immune functions such as chemotaxis, cellular invasion or lymphocyte proliferation. Somewhat paradoxically, modulation of mTOR activity, via rapamycin treatment, significantly increased the magnitude of the pro-inflammatory response, both in the target organ and peripherally, notably the spleen.

mTOR plays a pivotal role in determining the outcome of parasite antigen recognition by CD8^+^ T cells, because it functions as a principal sensor and integrator of the nutrient and energy status. In the same murine model of ECM, Mejia et al. [17] reported that dietary restriction during infection was associated with reduced mTORC1 (mechanistic Target of Rapamycin Complex 1) activity in T cells and resulted in protection against the onset of disease. Rapamycin treatment also inhibited mTORC1 and prevented ECM pathology. Together these studies support the mTOR pathway as a potential target for adjunctive therapeutic strategies in cerebral malaria treatment.

## mTOR: A therapeutic target for cerebral parasitosis?

Disruption of the BBB combined with neuro-inflammation is a hallmark of infection with human protozoan parasites clinically presenting with cerebral pathology [18]. Therefore, we are suggesting that targeting the mTOR pathway would represent a novel approach for the treatment of cerebral parasitosis. In addition, rapamycin is approved for use in humans (currently prescribed for some cancer patients and organ transplant patients), making it an appealing choice of therapeutic strategy [19]. Although not widely investigated in the context of protozoan infection, we propose that there are sufficient indications in the literature to warrant an investigation into the potential use of rapamycin as a treatment for cerebral parasitosis.

A primary candidate for consideration must be human African trypanosomiasis (HAT), caused by the protozoa *Trypanosoma brucei gambiense* and *T. b. rhodesiense*. After infection through the bite of the tsetse fly, parasites initially disseminate in the blood and lymphatic systems. As infection progresses, parasites penetrate into the central nervous system (CNS) initiating the meningoencephalitic stage of infection, a critical step in the progression of disease [20]. Invasion of the CNS by trypanosomes is not related to the level of parasitemia but is dependent on the host immune response and facilitated by T cells. In particular, a Th1 immune response increases trypanosome neuroinvasion; in the absence of interferon IFN-γ and T cells, parasite entry into the brain parenchyma is greatly reduced [21]. CD4^+^ T cells have been proposed to be the principal source of IFN-γ in *T. brucei-*infected mice, while CD8^+^ T cells have been associated with mortality, with CD8^−/−^ mice showing prolonged survival following infection compared to wild type mice [21]. Such a central role for T cells in the mediation of trypanosome neuroinvasion would support a possible therapeutic application for rapamycin. Of interest, daily administration of minocycline, a tetracycline antibiotic, to *T. brucei* infected mice reduced trypanosome CNS invasion [22]. This antibiotic displays a direct effect on T cells, preventing activation and transmigration, an outcome that was proposed as the mechanism impeding the movement of trypanosomes into the brain parenchyma. The impact of minocycline treatment was specific to the CNS, as the growth of *T. brucei* and the levels of cytokines in the spleen were unaffected. This study strongly supports the possibility that the use of rapamycin to target T cell activation would prevent the cerebral parasitosis associated with *T. brucei* infection.

*Acanthamoeba* are the causative agents of granulomatous amoebic encephalitis, a fatal disease of the CNS that, primarily presents in immune compromised patients [23]. The mechanisms by which *Acanthamoeba* breaches the BBB are complex but appear to involve both parasite proteases and host proinflammatory immune responses. Combined, these mediate increased permeability and apoptosis of brain endothelial cells, which disrupts the BBB and permits CNS invasion by the parasite [24]. Of relevance to our hypothesis is the observation that programmed cell death of brain endothelial cells mediated by *Acanthamoeba* is dependent on the activation of phosphatidylinositol 3-kinase (PI3K; [25]). Considering that mTOR is activated by p-Akt downstream of PI3K, it is likely that the administration of rapamycin would control the protozoan induced apoptosis of endothelial cells and thus block the movement of parasites into the brain. Indeed, rapamycin has been shown to inhibit programmed cell death induced by HIV infection, paclitaxel, UV irradiation and TNF [25].

It is estimated that up to 50% of the world’s population is infected with *Toxoplasma gondii* and that even though many people harbour dormant brain cysts which contain the slowly dividing bradyzoite stage of the parasite, most immune competent people are asymptomatic [26]. However, when an individual’s immune system is compromised the encysted bradyzoites covert to the tachyzoite stage, which in the brain results in a recrudescence of acute infection leading to toxoplasmic encephalitis (TE), a debilitating manifestation of the infection that can lead to severe and often life threatening meningitis [27]. An important step leading to TE is during the acute stage of infection, when the tachyzoite breaches the BBB, allowing dissemination to the brain parenchyma. Unlike ECM or HAT, there appears to be no role for T cells in the movement of *T. gondii* into the CNS. Instead, the most recent study suggests that this parasite compromises the blood brain barrier by invading, replicating in and then lysing brain endothelial cells. [28]. Importantly, activation of the mTOR pathway within cells was shown to be critical to support parasite expansion [29]. The correlation between increased mTOR activity and increased rapamycin-sensitivity of cell cycle progression in *T. gondii* infected cells therefore supports the application of rapamycin as a therapeutic approach to prevent parasite replication within brain endothelial cells and thus subsequent lysis and movement of the parasite into the CNS.

Numerous other neuropathogenic protozoa interact with the blood-brain barrier for the establishment of CNS infections [2]. The elucidation of a role for mTOR in the core processes of blood-brain barrier destruction for these additional pathogens could establish rapamycin as a novel therapeutic strategy to combat cerebral pathology caused by these protozoa.

## Targeting mTOR: A double pronged approach to regulate host and parasite metabolism

Given the critical role for mTOR signaling in cell metabolism it is perhaps unsurprising that Tor signaling is emerging as a functional pathway in the regulation of protozoan growth and proliferation. This implies that in addition to inhibiting the degradation of the BBB, targeting the parasite’s TOR pathway might directly impact the development of protozoan parasites and thus attenuate the pathogenesis during infection.

Initial support for this notion came from the observation that the liver-stage, asexual and sexual intraerythrocytic-stage of *P. falciparum* was inhibited by Torin2, the mTOR ATP-competitive kinase inhibitor [30]. However, identification of putative *Plasmodium* binding partners suggested that the inhibitory effect of Torin2 was not due to a targeted effect on the TOR pathway [30]. Instead, Torin2 was shown to interact with a number of proteins involved in the parasite’s metabolic pathways, including a putative nutrient transporter, a phosphoribosyl pyrophosphatase synthetase and an aspartate carbamoyltransferase [31]. More recently, mass spectrometry analysis of Torin-treated parasites identified a rapid and selective reduction in hemoglobin derived peptides, which indicated that Torin2 may be mediating its inhibitory effect by inhibiting essential hemoglobin catabolism [32]. Moreover, to date, no mTOR homologs have been identified in the genomes of *Plasmodium* parasites, which further negates the possibility that rapamycin could have a direct effect on the growth and proliferation of the parasite. Indeed, rapamycin treatment of mice infected with *Plasmodium berghei* resulted in elevated peripheral parasitemia [14].

Although also classified as protozoan, the trypanosomatids are phylogenetically quite distinct from the apicomplexan plasmodium parasites [33]. Indeed, analysis of the genomes of the trypanosomatid parasites *Leishmania major* and *T. brucei* has revealed the presence of two conserved signalling complexes, TOR1 and TOR2 [34–37], whose functions appear analogous to that described for mammalian TORs that mediate essential functions for cell growth. Accordingly, either depletion *T. brucei* mTOR2 or rapamycin exposure (which prevents tbTOR2 formation) resulted in aberrant cell morphology, impaired endocytosis, and blocked cytokinesis [36]. Similarly, the inability to general homozygous knockouts of *L. major* TOR1 or TOR2 supports essential roles in the survival of the parasite [34]. In contrast, *L. major* parasites deficient in a third TOR (TOR3) showed normal morphology. They were however, unable to survive or replicate in macrophages in vitro, or to induce pathology or establish infections in mice in vivo, which suggests an important role in the virulence of the parasite. Of interest, virulence of *Leishmania* parasites has been associated with the presence of sequence polymorphisms in other components of the mTOR pathway, with a mutation in a GTPase enzyme, shown to contribute to the attenuation of the cutaneous strain of *L. donovani* in visceral infection [38].

Thus, targeting the TOR pathway presents an opportunity for the design of anti-parasite agents for some protozoan parasites. As our knowledge and understanding of the genomes and proteomes of protozoans are expanded, it will be important to determine the level of requirement for TOR and other elements of the TOR pathway on parasite growth, as this will determine the potential effectiveness of rapamycin to inhibit both parasite proliferation and host immune responses.

## Conclusions

The observations described support the possibility that strategically targeting mTOR could influence the immune-mediated clinical cerebral outcomes of host-protozoan interactions and additionally act to limit protozoan replication. However, this may not always be the case. For example, after internalization, the human *Leishmania* protozoan secretes a protease (GP63) which cleaves mTOR, thus removing regulation of the translational repressor 4E-BP1, resulting in the promotion of parasite proliferation within cells. Consistent with these observations, rapamycin induced the activation of 4E-BP1, which increased the level of parasitic replication [39]. Despite preventing ECM when administered day 1 or day 4/5 post-infection, treatment with rapamycin at day 1 was associated with earlier death of the animals from hyperparasitaemia caused by an indirect effect on the mechanisms that control the parasite growth via the adaptive immune response [14]. Thus the differential host, protozoan-specific and disease phase-specific functions of mTOR must be more fully understood to optimize timing and parasite targets to determine whether it is realistic to target for therapeutic intervention. While clearly much more investigative work is required, we believe that the known mechanisms by which protozoan parasites traverse the blood brain barrier [40] provide sufficient support to our hypothesis that targeting mTOR represents a novel strategy in the treatment of cerebral parasitosis.

## Abbreviations

BBB: Blood brain barrier
CNS: Central nervous system
ECM: Experimental cerebral malaria
HAT: Human African trypanosomiasis
IFN: Interferon
mTOR: Mammalian target of rapamycin
mTORC1: Mechanistic Target of Rapamycin Complex 1
PRBC: Parasitised red blood cells
TE: Toxoplasmic encephalitis
